# Macro-Climatic Distribution Limits Show Both Niche Expansion and Niche Specialization among C_4 _Panicoids

**DOI:** 10.1371/journal.pone.0151075

**Published:** 2016-03-07

**Authors:** Lone Aagesen, Fernando Biganzoli, Julia Bena, Ana C. Godoy-Bürki, Renata Reinheimer, Fernando O. Zuloaga

**Affiliations:** 1 Instituto de Botánica Darwinion (CONICET-ANCEFN), Labarden 200, San Isidro, B1642HYD, Buenos Aires, Argentina; 2 Departamento de Métodos Cuantitativos y Sistemas de Información, Facultad de Agronomía, UBA, Buenos Aires, Argentina; 3 Instituto de Agrobiotecnologia del Litoral, Santa Fe de la Vera Cruz, Santa Fe, Argentina; Clemson University, UNITED STATES

## Abstract

Grasses are ancestrally tropical understory species whose current dominance in warm open habitats is linked to the evolution of C_4_ photosynthesis. C_4_ grasses maintain high rates of photosynthesis in warm and water stressed environments, and the syndrome is considered to induce niche shifts into these habitats while adaptation to cold ones may be compromised. Global biogeographic analyses of C_4_ grasses have, however, concentrated on diversity patterns, while paying little attention to distributional limits. Using phylogenetic contrast analyses, we compared macro-climatic distribution limits among ~1300 grasses from the subfamily Panicoideae, which includes 4/5 of the known photosynthetic transitions in grasses. We explored whether evolution of C_4_ photosynthesis correlates with niche expansions, niche changes, or stasis at subfamily level and within the two tribes Paniceae and Paspaleae. We compared the climatic extremes of growing season temperatures, aridity, and mean temperatures of the coldest months. We found support for all the known biogeographic distribution patterns of C_4_ species, these patterns were, however, formed both by niche expansion and niche changes. The only ubiquitous response to a change in the photosynthetic pathway within Panicoideae was a niche expansion of the C_4_ species into regions with higher growing season temperatures, but without a withdrawal from the inherited climate niche. Other patterns varied among the tribes, as macro-climatic niche evolution in the American tribe Paspaleae differed from the pattern supported in the globally distributed tribe Paniceae and at family level.

## Introduction

Grasses originated as tropical understory species, adapted to humid and shaded conditions, but have since developed into one of the most widespread, versatile, and species rich plant families on earth [1–3]. Grasses have reached all continents and adapted to all terrestrial ecosystems [4], yet niche evolution and the functional traits that underlie climatic range expansion in grasses are poorly understood [2,5]. Macroecological analyses that combine species distributions with phylogeny and GIS-based climate data have uncovered aspects of niche evolution in a wide range of taxa [6,7]. Within grasses, macroecological niche studies are presently aiming to pinpoint where new functional traits may have been acquired, as well as examining the correlation between putative key innovations and niche evolution [3,8,9].

C_4_ photosynthesis is considered one of the key innovations that allowed grasses to radiate in warm and open habitats [1,10]. The C_4_ pathway has evolved independently more than 60 times among angiosperms and 22–24 times within the grass family [11,12]. C_4_ photosynthesis is a syndrome, which requires modification of several genes, inducing both anatomical and biochemical modifications of the ancestral C_3_ pathway [1,10,13]. Although C_4_ photosynthesis is energetically more expensive than C_3_ photosynthesis, it nearly eliminates photorespiration, which reduces the productivity of the C_3_ pathway in CO_2_-depleted, hot, and water-stressed habitats [10]. By reducing photorespiration, the C_4_ syndrome is more productive in a wide range of tropical and subtropical habitats. However, the syndrome is less competitive at low temperatures where photorespiration declines, which may explain why C_4_ plants are rare in cold climates [14]. C_4_ grasses dominate all tropical and subtropical savannahs and constitute most of the grass flora in warm arid regions with summer rain [15]. At a global scale the C_4_ syndrome is found in less than 3% of all plant species but these are estimated to produce nearly a quarter of earth’s primary production [10,16]. Several key crops are C_4_ grasses (e.g., maize and sugarcane), but, on the other hand, many weeds and invasive species are C_4_ grasses too [17]. Understanding the evolution and ecological advantage of the C_4_ syndrome is therefore crucial not only for grass evolution, but for a wide range of disciplines including crop management as well as the history and ecology of tropical grasslands and their response to climate changes.

Long standing efforts to understand the biogeography of C_4_ grasses have described and quantified local and global diversity patterns of C_4_ species (see [15] and references therein). More recently, macroecological studies have aimed to quantify niche evolution in C_4_ grasses by comparing macro-climatic distribution data between C_3_ and C_4_ grasses in a phylogenetic context [3,8,18]. The most comprehensive of these analyses [3] found consistent differences in the mean values of annual rainfall and seasonality among closely related C_3_ and C_4_ species, but surprisingly, no differences among the temperature variables. The mean precipitation values of the C_3_ species were high enough to maintain a closed canopy vegetation, while the values of the C_4_ species were slightly lower and more seasonal corresponding to open vegetation. Macroclimatic data therefore seems to support that a change from C_3_ to C_4_ photosynthesis in grasses correlates with a habitat shift from the tropical understory to the tropical savannah system, and that C_4_ species occupy drier regions than their C_3_ relatives but not necessarily warmer ones [3].

Studies on macro-climatic niche evolution in grasses have, however, only examined species mean values not climatic limits. Some of the reasons for this are that public available distribution databases are error-prone and possible incomplete, which complicate obtaining reliable distribution limits in global data sets [19], however mean values are less affected. Yet, the niche concept as well as large scale biogeographic patterns are based on distributional limits [7]. Niches can expand, contract, or shift [20], and these changes bear different implications on what may be considered the evolutionary and ecological advantages of the C_4_ syndrome. The evolution of C_4_ photosynthesis may either correlate with a niche specialization or an expansion, depending on whether both or only a single climatic extreme changes position; in the latter case, C_4_ species become generalists that succeed in a wide range of habitats including those occupied by their closest C_3_ relatives. Recent analyses within the grass species *Alloteropsis semialata* (R. Br.) Hitchc. that contains both C_4_ and non-C_4_ genotypes suggest that a niche expansion may be the initially response to a change in the photosynthetic pathway, while niche specialization is a delayed response that requires speciation within the new habitat [21]. When macro-climatic distribution data is used to explore niche evolution, both of these niche changes may alter the mean values of the C_4_ species, but the patterns can be distinguished if the climatic extremes are compared; this is the aim of the present study.

C_4_ photosynthesis has evolved several times within grasses but only among the tropical and subtropical subfamilies gathered in the PACMAD clade (subfamilies Panicoideae, Arundinoideae, Chloridoideae, Micrairoideae, Aristidoideae, and Danthonioideae, [12]). All but five of the photosynthetic transitions are found in the subfamily Panicoideae sensu [12], which offers a rare opportunity to compare several parallel origins of the C_4_ syndrome among closely related species. The three main Panicoideae tribes comprise approximately half of the species in the PACMAD clade and nearly 30% of all grasses [12].

Here we explore whether the C_3_→C_4_ transitions correlate with a general pattern of macro-climatic niche evolution in the subfamily Panicoideae, using available distribution records to infer climatic extremes of three variables: aridity (AI), mean temperature of the warmest quarter (MTWQ), and mean temperature of the coldest quarter (MTCQ). We analysed the response both at subfamily level and within the subtribes Paspaleae and Paniceae that contain most of the C_3_→C_4_ transitions within Panicoideae [12]. For each variable, we explored whether C_4_ photosynthesis correlates with niche stasis, niche expansions, or a full niche changes.

## Materials and Methods

Most studies that analyse macro-climatic niche evolution in a phylogenetic context rely on the taxon sampling available for phylogenetic analyses. However, within Panicoideae (~3560 spp.) the two tribes that contain most of the C_3_→ C_4_ transformations, Paspaleae and Paniceae, include ~2170 species [22] among which less than 20% are available for phylogenetic analyses (see Phylogenetic Analyses below). The ratio of C_3_ to C_4_ species is ~1/5 within the two tribes but ~1/2 in the phylogenetic data set. This bias probably arose because panicoids have been sampled to address phylogenetic relationships above generic level, and not to analyse niche evolution. Sampling efforts have concentrated on the polyphyletic genus *Panicum*; where most of the newly segregated genera consist of C_3_ species. In contrast, the major C_4_ genera are severely undersampled in Panicoideae. The distribution data available through the Global Biodiversity Information Facility GBIF (www.gbif.org) is more complete. GBIF contains data for ~44% of the species in the two tribes and the C_3_/ C_4_ ration is ~1/3. The distribution data set is consequently more complete, and we use this for comparing climatic extremes within Panicoideae. However, DNA sequences are lacking for more than half of the species in this data set. To place these manually within the phylogeny, several nodes had to be collapsed, as the monophyly of various genera largely remain untested.

### Distribution and climate data

We downloaded all georeferenced species from the subfamily Panicoideae through GBIF (accessed November 2011 to December 2013). All species names were validated or synonymized according to the Catalogue of New World grasses ([23] updated at www.tropicos.org) or the Plant List (www.theplantlist.org). Intermediate C_3_-C_4_ species and species with five or fewer georeferenced locations were excluded. A total of 1307 species (nearly 40% of the subfamily) and more than 450.000 locality records were included in the final distribution analyses.

Because publically available distribution databases are error-prone and potentially incomplete [19], studies on macro-climatic niche evolution mostly examine species mean values not climatic limits. One potential problem is that the available records may or may not capture the geographic and/or macro-climatic ranges of the species (see [19]). However, grasses are economical important and well collected in many areas. Grasses may therefore be one of the better candidates for a comparative study on climatic extremes. Within Panicoideae, the macro-climatic minimum values, for the large majority of the species, fall in North America, southern South America, Australia, and southern Africa. Because grasses are economically important in these regions, they are well collected, and the records are available through GBIF. The maximum values fall in tropical regions of America, Africa, and Asia–where grasses are less collected. This bias should, however, affect C_3_ and C_4_ species alike. Rarefaction plots of the data are found in S1 Fig Rarefaction.

Temperature variables were extracted from BioClim (http://www.worldclim.org [24]) at a spatial resolution of 2.5 arc-minutes while aridity data was extracted from CGIAR-CSI at a spatial resolution of 30 arc-seconds (http://www.cgiar-csi.org).

**Fig 1:**
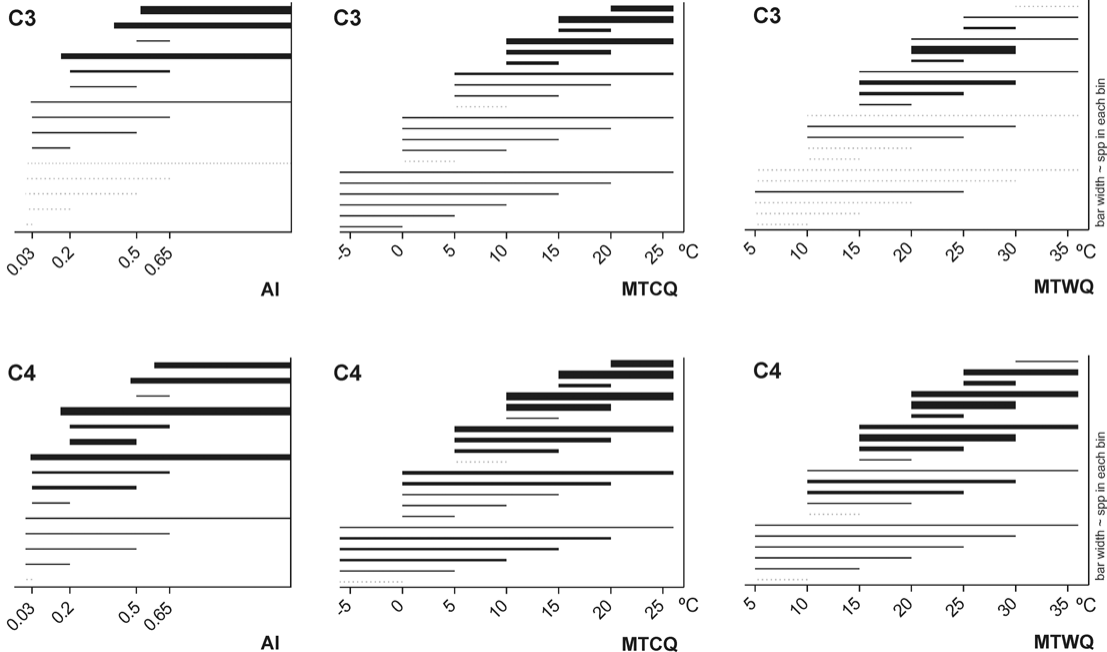
Macro-climatic ranges of the 1307 panicoid species from the climate data set including 237 C_3_ and 1070 C_4_ species ignoring phylogenetic relationships. The line widths of the bars are proportionate to the number of species in each bin. All quantitative analyses treated the Aridity Index (AI) and the Mean Temperature of the Coldest and Warmest Quarters (MTCQ and MTWQ) as continuous characters, but the variables are here binned for illustrative purposes. Following the United Nations Environment Programme we use the AI categories: <0.03 hyper arid, 0.03–0.2 arid, 0.2–0.5 semi-arid, 0.5–0.65 dry sub-humid, >0.65 humid. Following the Köppen climate classification, the limit between tropical and subtropical climates falls at approx. 18ºC.

Minimum and maximum values were extracted for each climatic variable for each species based on GBIF distribution data. We excluded 5% of the records at each extreme, to avoid distortion from misidentified species, spatially-imprecise georeferences, errors caused during digitalization of the herbarium specimens etc. The final climate data set is found in S1 Table Climate Data Set. The macro-climatic ranges contained in the climate data set are shown for C_3_ and C_4_ species in Fig 1.

#### Temperature

We explore whether the different temperature response of C_3_ and C_4_ photosynthesis affects the distribution of C_3_ and C_4_ panicoids, by analysing climatic extremes of both the ‘Mean Temperature of Warmest Quarter” and the ‘Mean Temperature of Coldest Quarter”. We based all quantitative analyses on continuous values but used bins of 5°C for graphic and descriptive purposes. According to the Köppen climate classification (see [25]), the limit for tropical climate coincides with a year round average temperature of 18°C or higher. In the present study, the limit for tropical climate mainly falls in the 15°-20°C bin except in Australia and Northern Africa where it falls in the 20°C-25°C because arid climate (like central Australia and Sahel/Sahara) is not considered tropical in the Köppen climate classification.

#### Aridity

Compared to the C_3_ pathway, the C_4_ syndrome has an inherited higher water use efficiency, and there has been much discussion in the literature as to whether the C_4_ syndrome is an adaptation to arid environments or merely improves the conditions for adapting to these (see [15,26] and references therein). Variables related to precipitation are therefore included in all C_4_ niche analyses. Because aridity is related to both rainfall and temperature, we compared climatic extremes among C_3_ and C_4_ species using the aridity index (AI = Mean Annual Precipitation/Mean Annual Potential EvapoTranspiration) extracted from the CGIAR-CSI Global Aridity and Global PET Geospatial Database [27]. We based all quantitative analyses on continuous values but used the categories established by the United Nations Environment Programme for graphic and descriptive purposes: Aridity Index (AI): <0.03 Hyper Arid, 0.03–0.2 Arid, 0.2–0.5 Semi-Arid, 0.5–0.65 Dry sub-humid, >0.65 Humid [27]. Grasslands (incl. savannahs and shrublands) are found in all categories except in hyper arid regions, but grasslands become the dominant biome in semi-arid regions while desert and forest dominate drier or more humid regions respectively [28,29].

### Phylogenetic analyses

We searched GenBank for sequences of *ndhF*, *rbcL*, and *trnL* for all available species of Panicoideae. The matrix included a total of 487 species of which 412 belong to one of the two tribes Paspaleae and Paniceae. We assembled the matrix with the program GB-to-TNT [30] calling Mafft for the alignment step [31]. Parsimony analyses were done using the program TNT ver. 1.1 [32], treating gaps as missing data.

Bayesian inferences of phylogenetic trees were done with MrBayes 3.1 [33] through the CIPRES portal [34]. Partitions were allowed to evolve under different models according to the JModel test 2.0 [35], using the GTR + I + G substitution model for *ndhF* and HKY +I +G for *rbcL* and *trnL*. Two different analyses, each of four parallel chains, were run for 20 736 000 generations, sampling a tree each 1000 generations and with a burn-in period of 2 500 000. The convergence of the MCMC (Markov Chain Monte Carlo) run and the adequacy of the burn-in length were confirmed using the program tracer v1.6.0 [36].

### Analyses of the climate data set

To analyse general trends within Panicoideae, we tested whether the upper and lower climate extremes differed among C_3_ and C_4_ species using Phylogenetic Generalized Least Squares (PGLS) regression [37,38]. PGLS regressions were done using the program R [39] and the package nlme [40], with correlation structures calculated from phylogenetic trees using the package ape [41]. For an input tree, we used a collapsed working phylogeny [42] including all 1307 taxa for which climate data was available. Branch lengths and topology were obtained from the Bayesian analyses, however, in the working phylogeny where more than half of the terminals lacked DNA sequence data, we only retained nodes that were both well supported and well sampled in recent phylogenetic studies (Table 1). The terminal taxa without sequence data were pasted into these polytomies and were either assigned the mean branch length of the terminals in the 487 taxon phylogeny, or alternatively, we used the mean branch length among the terminals within each retained clade. We also analysed the 1307 taxon working phylogeny setting all branch lengths equal. For each input tree, we compared five models, one without phylogenetic correlation and four models with variations in the phylogenetic correlation: Brownian motion, λ of [43], g of [44], and ρ of [37], selecting the model with lowest AIC.

**Table 1:** Nodes retained in the working phylogeny used for the PGLS regressions. At suprageneric level, we retained nodes that appear in several published phylogenetic analyses. At generic level we retained nodes that were both well supported and well sampled in recent phylogenetic studies. Node number refers to subtribes in Figs 2–4 and S2 Fig Phylogenetic tree. Number of species refers to the number of species in each clade for which climate data was available (a total of 1307 species, marked in bold).

Taxa	nr node	nr sp	Photosynthesis	References
Chasmanthieae+Zeugiteae		**16**	C_3_	[12]
Chasmanthieae		6	C_3_	[12]
Zeugiteae		10	C_3_	[12]
Tristachyideae+Centotheceae+Cyperochloeae+Thysanolaeneae		**50**	C_3_ + C_4_	[12]
Tristachyideae		44	C_4_	[12]
Centotheceae+Cyperochloeae+Thysanolaeneae		6	C_3_	[12]
Gynerieae+Arundinelleae+Andropogoneae+Paspaleae+Paniceae		**1241**	C_3_ + C_4_	[12]
Arundinelleae+Andropogoneae+Paspaleae+Paniceae+Reynaudia		1240	C_3_ + C_4_	[12]
Arundinelleae+Andropogoneae		319	C_4_	[12]
Arundinelleae		13	C_4_	[12]
Andropogoneae		306	C_4_	[12]
Paspaleae		349	C_3_ + C_4_	[12,46]
Arthropogoninae+Otachyriinae		61	C_3_ + C_4_	[12]
Arthropogoninae	2	45	C_3_ + C_4_	[12,46]
Arthropogoninae p.p. unresolved		29	C_3_ + C_4_	
Apochloa		10	C_3_	[47]
Coleataenia		6	C_4_	[48]
Otachyriinae	1	16	C_3_ + C_4_	[49]
Anthaenantia		3	C_4_	[49]
Otachyriinae p.p.		13	C_3_	[49]
Paspalinae		288	C_3_ + C_4_	[12,46]
Paspalinae p.p. unresolved		24	C_3_	
Ocellochloa		10	C_3_	[50]
Renvoizea		6	C_4_	[47]
Streptostachys+Axonopus		50	C_3_ + C_4_	[12]
Streptostachys		1	C_3_	[46]
Axonopus		49	C_4_	[51]
Paspalum+Aakia+Anthaenantiopsis+Osvaldoa		198	C_4_	[52]
Paspalum		195	C_4_	[12,46]
Aakia+Anthaenantiopsis+Osvaldoa		3	C_4_	[52]
Paniceae		571	C_3_ + C_4_	[12,46]
Anthephorinae		72	C_4_	[12,46]
Boivinellinae	6	59	C_3_ + C_4_	[12,46]
Boivinellinae p.p. unresolved		29	C_3_	
Alloteropsis		4	C_4_	[53]
Echinochloa		21	C_4_	[12]
Parodiophyllochloa		5	C_3_	[54]
Neurachninae	4	17	C_3_ + C_4_	[12,46]
Ancistrachne+Calyptochloa+Cleistochloa		7	C_3_	[12]
Neurachne+Paraneurachne		7	C_3_ + C_4_	[55]
Thyridolepis		3	C_3_	[55]
Dichantheliinae	3	43	C_3_	[56]
Dichanthelium		41	C_3_	[56]
Adenochloa		2	C_3_	[56]
SubCladeB	5	47	C_3_	[12,46]
SubCladeC+Melinidinae+Panicinae+Cenchrinae		333	C_3_ + C_4_	[12,46]
SubCladeC	7	2	C_3_	[12,46]
Melinidinae+Panicinae+Cenchrinae		331	C_4_	[12,46]
Melinidinae+Panicinae		179	C_4_	[57]
Melinidinae		99	C_4_	[12,46]
Panicinae		80	C_4_	[12,46]
Cenchrinae		152	C_4_	[12,46]

To analyse distribution patterns at a lower taxonomic scale that may differ from the general trends [45], we repeated the PGLS regressions in the individual tribes Paspaleae and Paniceae, with branch lengths set as in the analyses at subfamily level.

## Results

### Phylogenetic analyses

We obtained the same topology as published in recent parsimony and Bayesian analyses (e.g., [12,46]). Also in agreement with earlier analyses, tribes and subtribes were monophyletic and mostly well supported, while the relationships among and within the subtribes were poorly resolved. The strict consensus tree and branch supports from the parsimony analysis are shown in S2 Fig Phylogenetic tree; names of tribes and subtribes follow [46] and [56]. Depending on the topology, there are 16–19 C_3_→C_4_ transitions within the Panicoideae. The major C_4_ clades are the tribe Andropogoneae, the genus *Paspalum* (tribe Paspaleae), and the subtribes Melinidinae, Panicinae, and Cenchrinae (tribe Paniceae). The C_4_ syndrome differs in anatomical details among the latter subtribes and is considered to have evolved independently in each [46].

### Analyses of the climate data set

**Fig 2:**
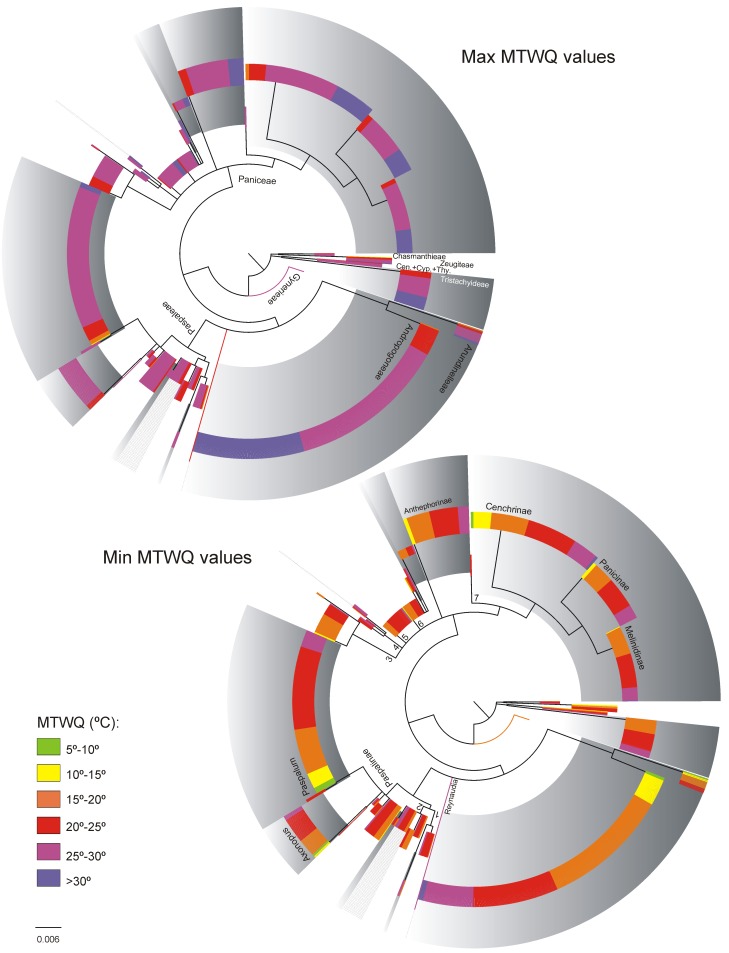
The climatic extremes of MTWQ in the 1307 panicoid grasses included in the climate data set. The topology shows the nodes retained in the working phylogeny used in the phylogenetic contrast analyses of the climate data set. The circle cladogram has been rooted in *Gynerium* for illustrative purpose, in the phylogenetic contrast analyses the cladogram was rooted as in S2 Fig Phylogenetic tree. Branch lengths were obtained from the Bayesian analyses, in the present tree; terminal taxa were assigned the mean branch length within each terminal clade (for details of the nodes see the 400 taxa cladogram, S2 Fig Phylogenetic tree). Clades with gray background colour are C_4_ clades. C_4_ species that do not form a clade are marked with gray lines (in Arthropogoninae and Neurachninae). Branch numbers refer to names of subtribes in Table 1. All quantitative analyses treated the MTWQ as a continuous character, but for illustrative purposes the values have been binned in the figure. Cen. = Centotheceae, Cyp. = Cyperochloeae, Thy. = Thysanolaeneae.

**Fig 3:**
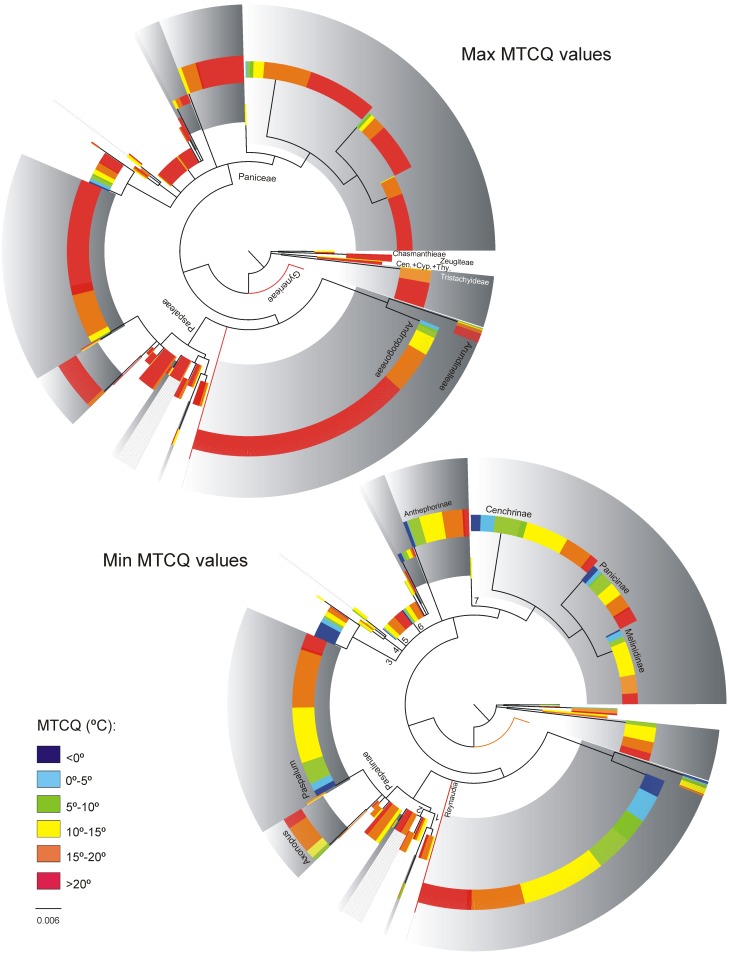
The climatic extremes of MTCQ in the 1307 panicoid grasses included in the climate data set. The topology shows the nodes retained in the working phylogeny used in the phylogenetic contrast analyses of the climate data set. The circle cladogram has been rooted in *Gynerium* for illustrative purpose, in the phylogenetic contrast analyses the cladogram was rooted as in S2 Fig Phylogenetic tree. Branch lengths were obtained from the Bayesian analyses, in the present tree; terminal taxa were assigned the mean branch length within each terminal clade (for details of the nodes see the 400 taxa cladogram, S2 Fig Phylogenetic tree). Clades with gray background colour are C_4_ clades. C_4_ species that do not form a clade are marked with gray lines (in Arthropogoninae and Neurachninae). Branch numbers refer to names of subtribes in Table 1. All quantitative analyses treated the MTCQ as a continuous character, but for illustrative purposes the values have been binned in the figure. Cen. = Centotheceae, Cyp. = Cyperochloeae, Thy. = Thysanolaeneae.

**Fig 4:**
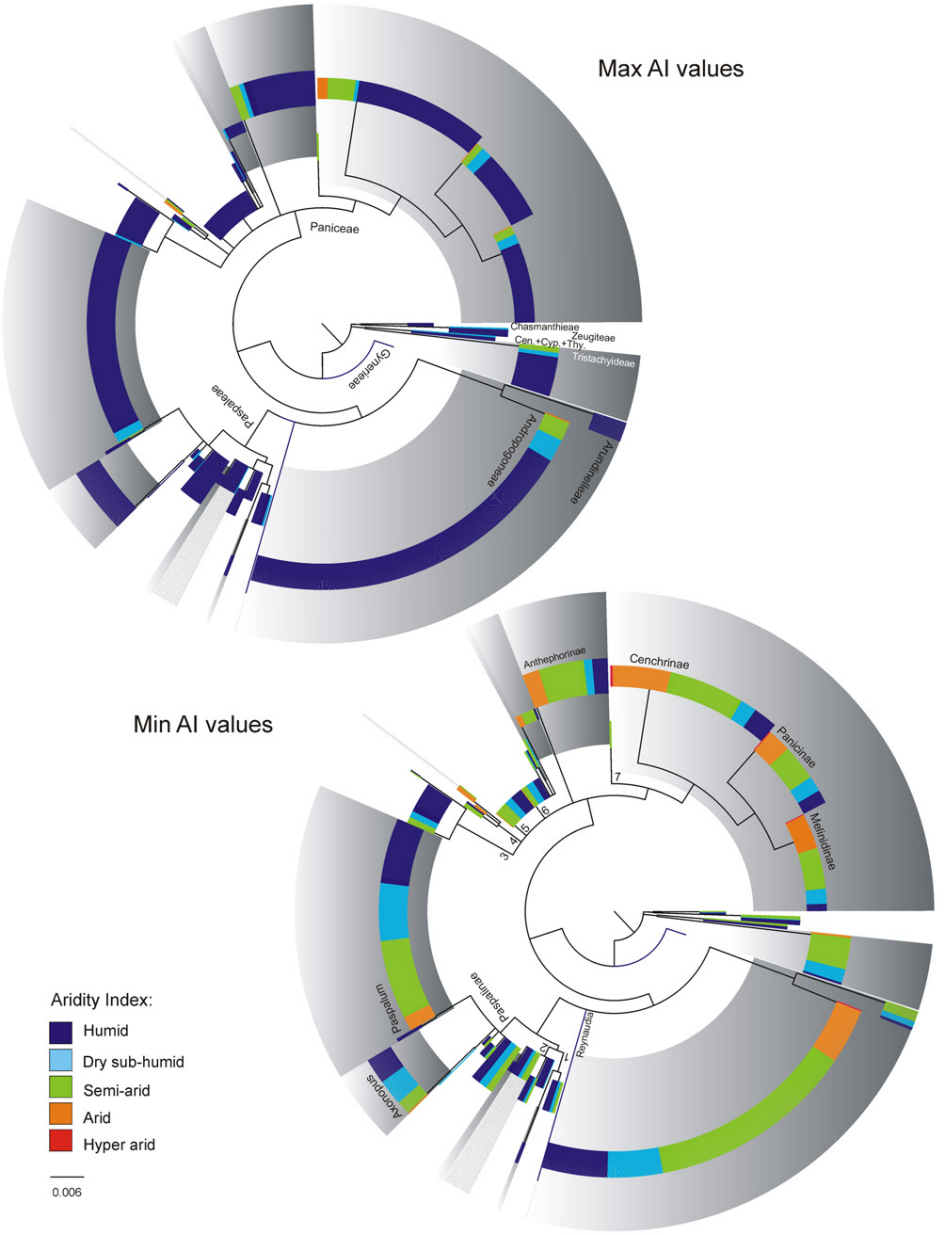
The climatic extremes of the Aridity Index in the 1307 panicoid grasses included in the climate data set. The topology shows the nodes retained in the working phylogeny used in the phylogenetic contrast analyses of the climate data set. The circle cladogram has been rooted in *Gynerium* for illustrative purpose, in the phylogenetic contrast analyses the cladogram was rooted as in S2 Fig Phylogenetic tree. Branch lengths were obtained from the Bayesian analyzes, in the present tree; terminal taxa were assigned the mean branch length within each terminal clade (for details of the nodes see the 400 taxa cladogram, S2 Fig Phylogenetic tree). Clades with gray background colour are C_4_ clades. C_4_ species that do not form a clade are marked with gray lines (in Arthropogoninae and Neurachninae). Branch numbers refer to names of subtribes in Table 1. All quantitative analyses treated the Aridity Index as a continuous character, but for illustrative purposes the values have been binned into the categories established by the United Nations Environment Programme: <0.03 hyper arid, 0.03–0.2 arid, 0.2–0.5 semi arid, 0.5–0.65 dry sub-humid, >0.65 humid. Cen. = Centotheceae, Cyp. = Cyperochloeae, Thy. = Thysanolaeneae.

Fig 1 shows the macro-climatic ranges of C_3_ and C_4_ species within the data set after eliminating 5% of the records at each climatic extreme. Figs 2–4 show the climatic extremes in the phylogenetic context used in the PGLS regressions. The results of the PGLS regressions are found in Table 2. In all cases the AIC criterion pointed to Grafen or Pagel as the best fitting model, but except for a single case (marked with an * in Table 2) none the alternative models with ΔAIC ≤10 contradicted the results shown in Table 2.

**Table 2:** Results from the phylogenetic contrasts comparing range limits between C_3_ and C_4_ species in the climate data set containing 1307 panicoid grasses. The PGLS regressions were performed at subfamily level (Panicoideae), and in the tribes Paspaleae and Paniceae. The model with lowest AIC value was selected as the best fitting model. P-values in bold indicate significant differences in means among C_3_ and C_4_ species. Branch lengths: 1: all branch lengths equal; 2: length of internal branches from Bayesian trees, length of terminal branches mean of all terminals in Bayesian trees; 3: length of internal branches as in 2, length of terminal branches mean of terminals in each individual clade of the Bayesian trees.

Clade	variable	Branch lengths	best-fitting model	estimated mean C_3_	estimated mean C_4_	C_4_ Relative to C_3_			P
Panicoideae	AI max	1, 2, 3	Grafen	1.59	1.28		-0.30	**0.0033**	
	AI min	1, 2, 3	Grafen	0.64	0.46		-0.18	**<0.0001**	
	MTCQ max	1, 2, 3	Grafen	21.00	22.22		1.22	0.1073	
	MTCQ min	1, 2, 3	Grafen	13.99	13.12		-0.87	0.4618	
	MTWQ max	1, 2, 3	Grafen	26.56	28.17		1.61	**<0.0001**	
	MTWQ min	1, 2, 3	Grafen	20.71	21.09		0.38	0.5756	
Paspaleae	AI max	1, 2, 3	Grafen	1.60	1.50		-0.10	0.3711	
	AI min	1	Pagel	0.72	0.63		-0.09	0.05*	
		2	Pagel	0.72	0.63		-0.09	0.0518	
		3	Pagel	0.72	0.63		-0.09	0.0528	
	MTCQ max	1, 2, 3	Grafen	22.18	22.8800		0.70	0.3606	
	MTCQ min	1, 2, 3	Grafen	16.82	14.21		-2.61	**0.045**	
	MTWQ max	1, 2	Grafen	25.60	27.17		1.57	**<0.0001**	
		3	Pagel	25.67	27.08		1.41	**<0.0001**	
	MTWQ min	1, 2, 3	Grafen	20.84	20.73		-0.11	0.9045	
Paniceae	AI max	1	Pagel	1.51	1.05		-0.46	**0.01**	
		2	Pagel	1.54	1.08		-0.46	**0.0206**	
		3	Pagel	1.51	1.08		-0.43	**0.025**	
	AI min	1, 2, 3	Grafen	0.62	0.37		-0.25	**<0.0001**	
	MTCQ max	1	Pagel	19.79	21.05		1.26	0.3731	
		2	Pagel	20.18	21.44		1.26	0.3896	
		3	Pagel	19.90	21.46		1.56	0.3038	
	MTCQ min	1	Pagel	11.96	11.64		0.32	0.8647	
		2	Pagel	12.09	12.01		-0.08	0.9654	
		3	Pagel	12.05	12.16		0.11	0.9561	
	MTWQ max	1, 2, 3	Grafen	27.04	28.43		1.39	**0.0105**	
	MTWQ min	1	Pagel	21.00	21.65		0.65	0.4286	
		2, 3	Grafen	20.42	21.32		0.90	0.3309	

*not significant under Grafen Delta AIC = 2.6.

#### Mean Temperature of Warmest Quarter

Consistent with the known global biogeographic pattern of C_4_ species, the majority of the C_4_ panicoids are found in regions where the MTWQ lie above 15°C, though several species reach areas with lower temperatures (Fig 1). The maximum MTWQ for most C_4_ species lie between 25°-35°C. The distribution of the C_3_ panicoids is similar to the one observed among C_4_ species, but very few C_3_ species appear in regions where the MTWQ reach above 30°C or below 15°C.

When analysing the climatic extremes in a phylogenetic context (Fig 2), MTWQ was the only climate variable that produced an invariable pattern at subfamily level and within both subtribes (Table 2). In all analyses the C_4_ species had significant higher maximum values compared to the C_3_ species, while the minimum values were indistinguishable.

#### Mean Temperature of Coldest Quarter

Most panicoids are found in regions where the MTCQ lie above 15°C, but both photosynthetic types reach areas with lower temperatures including regions with subzero MTCQ (Fig 1).

When analysing the climatic extremes in a phylogenetic context (Fig 3), the MTCQ maximum and minimum values of C_3_ and C_4_ species were indistinguishable at subfamily level and within the cosmopolite tribe Paniceae. However, within the American tribe Paspaleae, the minimum MTCQ values of the C_4_ species were significantly lower than the minimum values of the C_3_ species, while the maximum values were indistinguishable.

#### Aridity

Panicoid grasses are mainly found in semi-arid to humid climate, irrespectively of photosynthetic pathway (Fig 1). Most species have broad macro-climatic AI ranges, but some C_4_ species are restricted to semi-arid regions—a distribution pattern that is uncommon among the C_3_ panicoids. Several C_4_ panicoids furthermore reach into arid climate (occasionally hyper-arid), a pattern that is also uncommon among the C_3_ panicoids.

When analysing the climatic extremes in a phylogenetic context (Fig 4), the PGLS regressions found lower AI values in both climatic extremes among C_4_ species compared to C_3_ species (Table 2). These differences were significant in all analyses at subfamily level and within the tribe Paniceae. Within Paspaleae only the minimum values differed significantly from the values of the C_3_ species, and only in one of the analyses, while no differences were found among the maximum values (Table 2).

## Discussion

The present study supports that niche expansion into hotter climates is one of the main responses to a change from C_3_ to C_4_ photosynthesis among panicoid grasses. In fact, significantly higher MTWQ maximum values of the C_4_ species was the only ubiquitous response to C_4_ evolution within the subfamily Panicoideae (Table 2). A niche change into more arid climate was also supported at family level and within the cosmopolite tribe Paniceae but not in the American tribe Paspaleae.

Because the C_4_ pathway is more productive than C_3_ photosynthesis at high temperatures, a niche expansion of the C_4_ panicoids into warmer climates is to be expected, even if earlier phylogenetic contrast studies on grasses did not find support for the pattern [3]. In the subfamily Panicoideae, several species from all major C_4_ clades reach regions with MTWQ >30°C while few C_3_ panicoids reach temperatures this high (Fig 1). Within the American tribe Paspaleae, the C_4_ lineages reach slightly lower maximum MTWQ (Fig 2), but the values are still significant higher than the values among the related C_3_ species (Table 2). We did not find evidence for a macro-climatic niche specialization to hot climate among the C_4_ species. C_4_ panicoids that reach regions with high temperatures are also found areas where the MTWQ is similar to the temperatures occupied by the largely tropical C_3_ panicoids (Figs 1 and 2), and no differences could be detected in the minimum MTWC values (Table 2).

Expansion into arid regions and withdrawal from humid ones were the second most common response to C_4_ evolution in the subfamily, but it was only partly supported in the American tribe Paspaleae (Table 2). Differences in both minimum and maximum AI values were found both at family level and within the cosmopolite tribe Paniceae, while in Paspaleae, the PGLS regressions found significant differences in maximum AI values in a single analysis only and no differences in the minimum AI values among the C_3_ and C_4_ species (Table 2).

Panicoid species from arid regions mainly belong to the globally distributed tribes Andropogoneae (all C_4_) and Paniceae (Fig 4). The tribe Paniceae furthermore contains the Australian subtribe Neurachninae, which includes the only known C_3_ panicoids adapted to open dry habitats [58]. However, in the climate data set most of the Paniceae species that reach arid regions belong to the C_4_ subtribes Cenchrinae, Melinidinae, and Panicinae (see S1 Table Climate Data Set). In contrast to the arid adapted C_3_ species, there is little in the distribution pattern of the C_4_ species to suggest that these are drought resistant. Half of the C_4_ species that reach arid regions are annuals that may complete their life cycles under brief periods of rain, and several of the climatic wide-ranging perennials have been collected in microsites such as creeks and river banks in the drier part of their ranges. Most notable, comparative physiological studies of climatic wide-ranging and mesic C_4_ grasses as well as their C_3_ relatives show that the performance advantages of the C_4_ species are reduced or even lost under drought [59,60]. Although some of the C_4_ panicoids restricted to arid climates could prove to be drought resistant, most climatic wide-ranging C_4_ species may simply be microhabitat specialists that rely on efficient dispersal and high growth rates to maintain part of their populations within arid regions. Only field studies can determine whether the climatic wide-ranging C_4_ panicoids are generalists or simply very efficient microhabitat specialists. Being microhabitat specialists in the extreme part of their climate ranges, rather than dry adapted, would explain how climatic wide-ranging C_4_ species maintain their fitness to both inherited and new climatic conditions, and, if gene flow between populations is high enough, the general lack of species specialized to the latter [61,62].

While the higher water use efficiency of the C_4_ syndrome is considered to favour niche expansion of C_4_ species into warmer and/or drier climate, the lack of competitiveness at low temperature may hinder the C_4_ species from adapting to cold ones [14]. Nevertheless, in Panicoideae most subtribes include C_4_ species that reach regions with MTCQ below 0°C (Fig 3). Freezing tolerance during the dormant state may be relatively easy to acquire for both C_3_ and C_4_ species [14], but the C_4_ syndrome requires high daylight temperatures, to be competitive during the growing season [14]. Field studies show that C_4_ species from cold regions are restricted to warm microsites [14,63], which suggest that traits related to dispersal and recruitment, rather than cold adaptation of the C_4_ syndrome, maintain these edge populations.

The C_3_ pathway is not constrained by low temperatures [14], yet we found no evidence suggesting that C_3_ panicoids are more successful than C_4_ species in cold climates. None of the PGLS regressions found differences in the minimum MTWQ values among C_3_ and C_4_ panicoids, while the minimum MTCQ values differed within the American tribe Paspaleae where the C_4_ species reach regions with lower temperatures than the C_3_ species (Table 2). A large number of C_4_ species from a wide range of taxonomic groups are known to occur in regions with low winter temperatures and some of these tolerate occasional subzero temperatures during the growing season [14]. Within Paspaleae, the genus *Paspalum* in particular is known to include several high Andean species. However, while the C_3_ Paspaleae are restricted to regions where the MTCQ remain above 10°C (Fig 3), C_4_ species from all three Paspaleae subtribes can be found in areas with low winter temperatures in North and South America, and the expansion of C_4_ species into regions with low MTCQ is, in this study, supported as a general trend within the tribe (Table 2).

Although expansion of C_4_ species into regions with low winter temperatures is only supported in the tribe Paspaleae, C_4_ panicoids are diverse in regions with subzero MTCQ (Fig 3). In Paniceae both C_3_ and C_4_ lineage reach regions with subzero MTCQ and the minimum MTCQ for both C_3_ and C_4_ species in Paniceae were notably lower that in Paspaleae (Table 2). Most of the C_3_ panicoids that reach low temperatures do, however, belong to a single Nearctic radiation of the genus *Dichanthelium* (Dichantheliinae, see Fig 3 and S2 Fig Phylogenetic tree), which also includes the only C3 panicoids restricted to cold regions. Nearctic *Dichanthelium* species may, nevertheless, prove to be restricted by the same temperature requirements as the C_4_ species; their geographical northern limit concur with the C_4_ species, and in the climate data set *Dichanthelium* only appears in regions where the growing season temperatures reach the values reported for C_4_ species. Some phenological stages within the growing season may therefore show to have conserved niches within the Panicoideae and restrict the distribution of both C_3_ and C_4_ species, but phenological data must be included in niche studies to examine this. At present, the climate data set suggests that C_3_ panicoids are not more successful than their C_4_ relatives in cold climates and that more C_4_ than C_3_ panicoid lineages have reached cold regions. This is in agreement with recent ecophysiological studies that suggest C_4_ species have no intrinsic barrier to developing freezing tolerance, and that in some ecosystems the chance of developing freezing resistance may depend more on the capacity for drought resistance than on photosynthetic pathway [64,65].

Paspaleae constitutes the grass tribe with the highest number of closely related C_3_ and C_4_ lineages; it contains nearly half of the C_3_→ C_4_ transitions in Panicoideae, and includes the most species rich C_4_ genus of the grass family, *Paspalum*. Yet, in the tribe Paspaleae, the response to a change in the photosynthetic pathway differed from what was supported at subfamily level and within the cosmopolitan tribe Paniceae. Lundgren et al. [21] who studied the response of a transition in the photosynthetic pathway within the species *Alloteropsis semialata* (tribe Paniceae), found that C_4_ photosynthesis acted as a niche opener, which allowed the C_4_ specimens to occupy a wide range of new environments. Given enough time, speciation within the broader C_4_ niche could lead to specialization to some of these new environments. How taxonomic groups respond to a change in the photosynthetic pathway is therefore not only conditioned by the physiological advantages of the C_4_ syndrome but also by the genetic background of the C_3_ species that gave rise to a given C_4_ lineage [66,67], by the time since the transition [21], and by the availability, proximity, and extension of new niches [68].

The Paspaleae C_4_ species have expanded their niches into warmer—but not dryer–climates, and into regions with lower winter temperatures (Table 2). This distribution pattern supports that the common observed niche expansion of C_4_ grasses into arid macro-climates may be a delayed response, which only relates indirectly to C_4_ photosynthesis and requires further trait evolution [26]. The American tribe Paspaleae is most diverse in the Neotropics [23] where arid regions with summer rain are of relatively limited extension compared arid regions in Australia and Africa. Species from the older cosmopolite C_4_ grass subfamilies Chloridoideae and Aristidoideae are common in these arid parts of America, but within Paspaleae only a few C_4_ species reach arid sites (Fig 4). While the American continent is relatively humid, compared to Africa and Australia, the Andes chain provides a 7000 km long steep temperature gradient that runs through all of South American. The different response to C_4_ photosynthesis found within Paspaleae may simply reflect that several of the C_3_→ C_4_ transitions within Paspaleae seem to be of relatively young ages (see [10] and references therein), and that the composition and extensions of available niches in America differ from those found in other tropical continent.

In the climate data set (S1 Table), several closely related C_3_ and C_4_ Paspaleae species have nearly identical macro-climatic ranges and most of these are found in the Neotropical savannah system. Recent macroecological studies suggest that C_4_ species are closely related to C_3_ species from shaded habitats [3,18]. However, the Neotropical savannah system is a mosaic landscape with patches of open grassland, savannah, and gallery forests [69], and grasses from shaded or open habitats are sympatric at a macroecological scale (e.g., [70]). Field observations, not macroecological analyses, must therefore address whether the C_3_→C_4_ transitions correlate with habitat shifts within these savannahs. It should also be noted that several of the Neotropical C_3_ panicoids are open habitat species that occupy the well drained part of the savannah, some are fire resistant, and some are robust tussock grasses that may be locally dominant [71,72]. Which characters allowed the C_3_ species to invade open environments is unclear. Frequent shifts between open and shaded habitats happen in nearly all panicoid subtribes (see S2 Fig Phylogenetic tree), and functional traits related to leaf form, rather than photosynthetic pathway, have been found to correlate with such habitat shifts in subtropical grasses [73].

## Conclusions

In all, C_4_ panicoid conform to the known distribution patterns observed for C_4_ species at a global scale. C_4_ panicoids occupy significant hotter and dryer regions than their closest C_3_ relatives, and may also reach colder ones as long as the growing season temperatures are high enough. However, by comparing climatic extremes rather than diversity patterns or climatic values of centre populations, we found that these distribution patterns have been formed by a mixture of niche expansions, niche changes, and niche stasis among the C_4_ panicoids. Only niche expansion into hotter climate was a ubiquitous response to evolution of C_4_ photosynthesis within the subfamily, while the remaining patterns varied among subtribes and taxonomic level. This supports recent analyses suggesting that the ecophysiological advantages of the C_4_ syndrome act as a niche opener, which improve chance of survival after long distance dispersal [21]. Which new environments become colonized is then dependent both on the species inherited niche as well as available habitats and the history of chance dispersal within each C_4_ lineage.

## Supporting Information

S1 Fig Rarefaction. (PDF)

S2 Fig Phylogenetic tree. (PDF)

S1 Table Climate Data Set. (XLSX)
