# Supplementary material for: Macro-Climatic Distribution Limits Show Both Niche Expansion and Niche Specialization among C4 Panicoids
Source: PLoS One. 2016 Mar 7;11(3):e0151075. doi: 10.1371/journal.pone.0151075 (PMC4780779; doi:10.1371/journal.pone.0151075)
Supplement: S1 Fig — (PDF) [file pone.0151075.s001.pdf]

Rarefaction of the Panicoideae data set

For the rarefaction analysis, we selected three taxonomic groups with a variable number of specimens: a cosmopolite C_4 group (*Echinochloa*), an American C_4 group (*Paspalum*), and an African/American C_3 group (subcladeB).

	Nr of species	min nr of specimens	max nr of specimens
Echinochloa	21	5	4624
Paspalum	199	5	1664
subcladeB	59	5	1293

For each group, we ran 500 simulations for each of the following subsamples: 25%, 50%, 75%, and 90% of the specimens (the sampling was done without replacement). In each simulation we identified the maximum and minimum values of each species for the variables Aridity (AI), Mean Temperature of Coldest Quarter (MTCQ), and Mean Temperature of Warmest Quarter (MTWQ). The values were standardized (using values between 0 and 1) within each species, and means values were obtained for each of the 500 simulations (see Figure below).

The results show that with a subsample 50% of the available specimens, nearly all minimum and maximum values fell within the highest (or lowest) quartile. With 75% of the specimens subsampled all values fell very close to the maximum and minimum values identified for each species and used in the PGLS regressions.

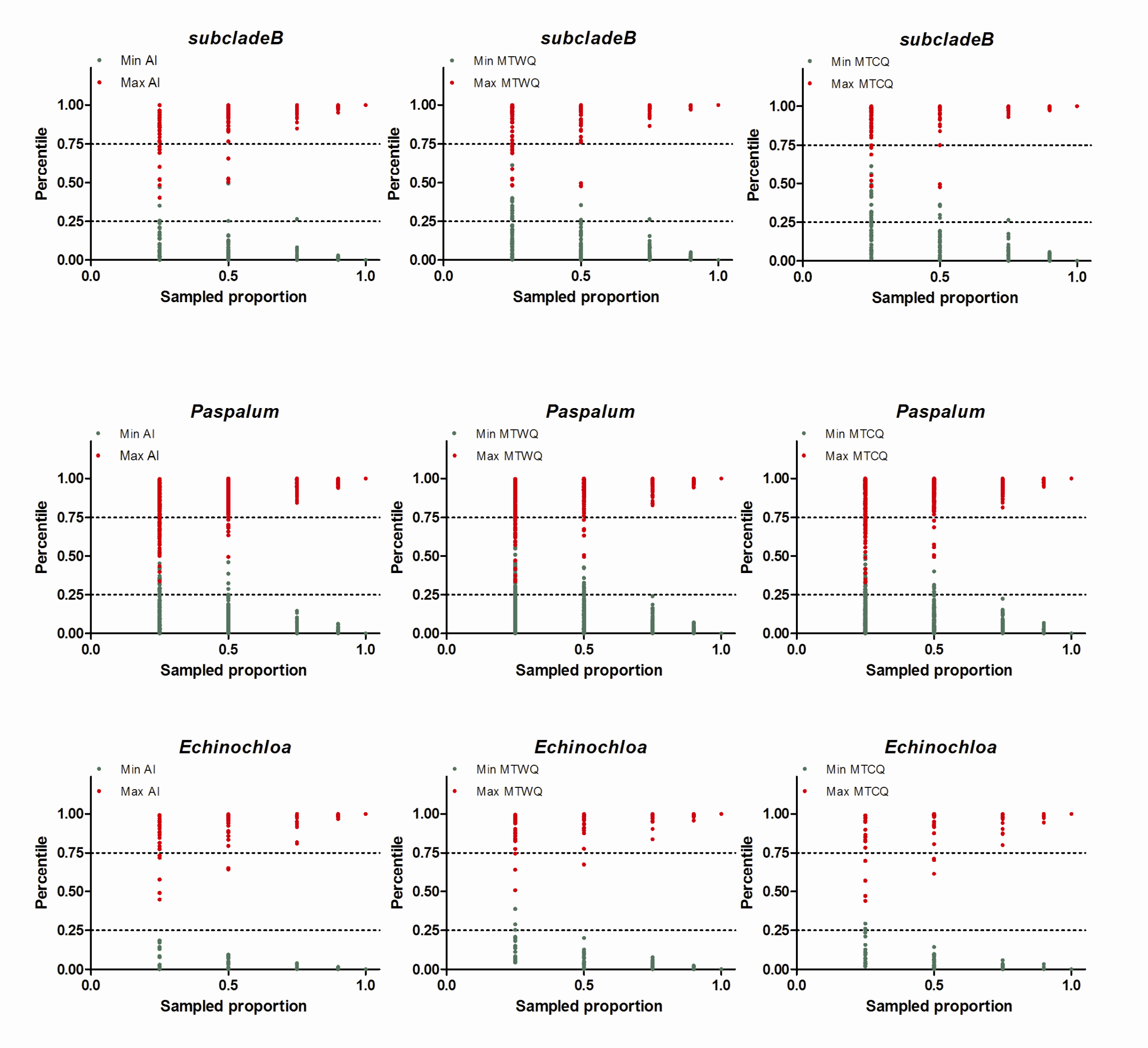

Rarefaction analyses for three selected taxonomic groups. Each point shows the mean minimum and maximum values of 500 simulations of all species within each taxonomic group, subsampling 25%, 50%, 75%, and 90% of the specimens in each species.
