# Supplementary material for: Macro-Climatic Distribution Limits Show Both Niche Expansion and Niche Specialization among C4 Panicoids
Source: PLoS One. 2016 Mar 7;11(3):e0151075. doi: 10.1371/journal.pone.0151075 (PMC4780779; doi:10.1371/journal.pone.0151075)
Supplement: S2 Fig — (PDF) [file pone.0151075.s002.pdf]

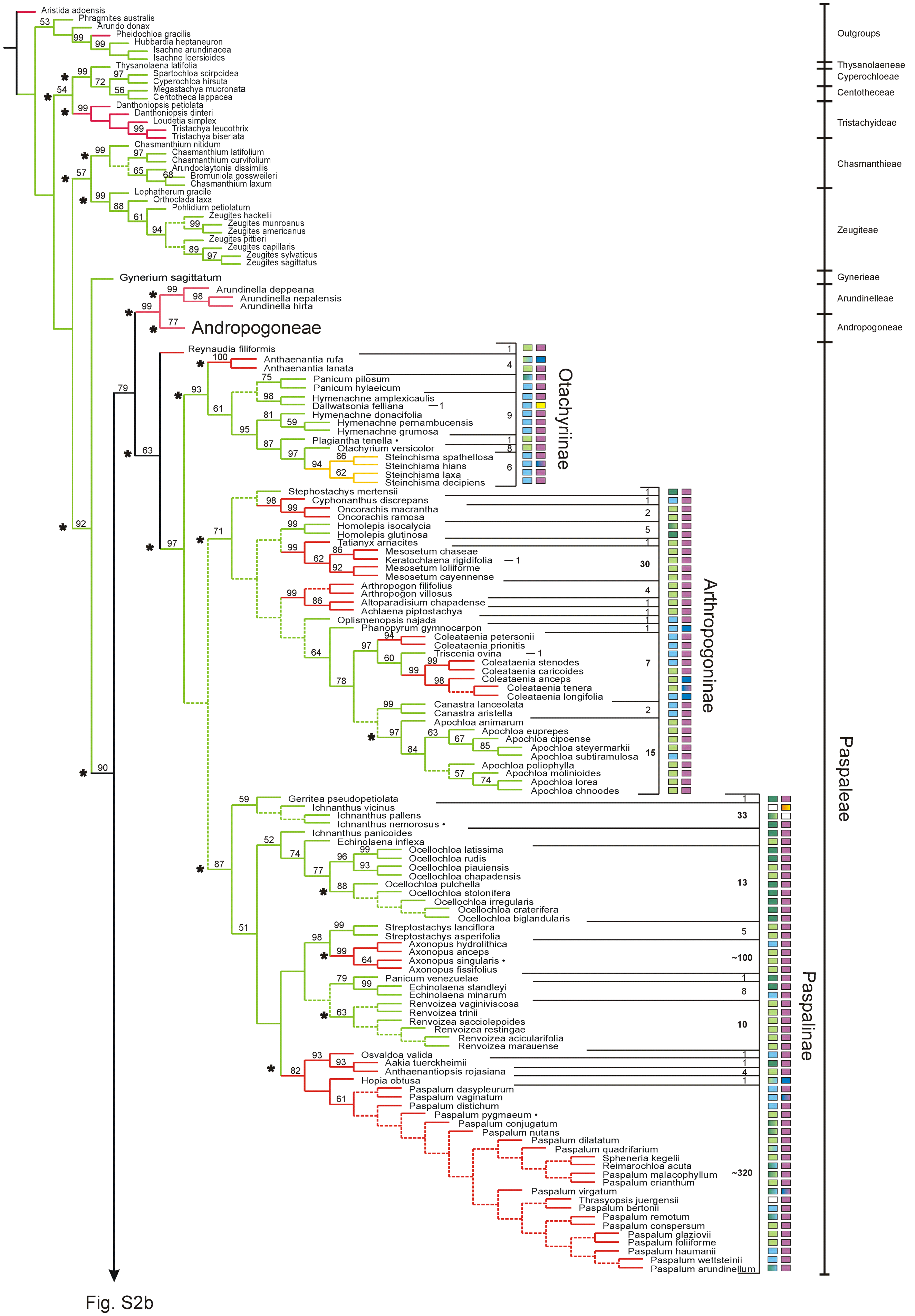
Aristida adoensis
Phragmites australis
Arundo donax
Pheidochloa gracilis
Hubbardia heptaneuron
Isachne arundinacea
Isachne leersioides
Thysanolaena latifolia
Spartochloa scirpoidea
Cyperochloa hirsuta
Megastachya mucronata
Centotheca lappacea
Danthoniopsis petiolata
Danthoniopsis dinteri
Loudetia simplex
Tristachya leucothrix
Tristachya biseriata
Chasmanthium nitidum
Chasmanthium latifolium
Chasmanthium curvifolium
Arundoclaytonia dissimilis
Bromuniola gossweileri
Chasmanthium laxum
Lophatherum gracile
Orthoclada laxa
Pohlidium petiolatum
Zeugites hackelii
Zeugites munroanus
Zeugites americanus
Zeugites pittieri
Zeugites capillaris
Zeugites sylvaticus
Zeugites sagittatus
Gynerium sagittatum
Arundinella deppeana
Arundinella nepalensis
Arundinella hirta
Andropogoneae
Reynaudia filiformis
Anthaenantia rufa
Anthaenantia lanata
Panicum pilosum
Panicum hylaeicum
Hymenachne amplexicaulis
Dallwatsonia felliana
Hymenachne donacifolia
Hymenachne pernambucensis
Hymenachne grumosa
Plagiantha tenella •
Otachyrium versicolor
Steinchisma spathellosa
Steinchisma hians
Steinchisma laxa
Steinchisma decipiens
Otachyriinae
Stephostachys mertensii
Cyphonanthus discrepans
Oncorachis macrantha
Oncorachis ramosa
Homolepis isocalycia
Homolepis glutinosa
Tatianyx arnacites
Mesosetum chaseae
Keratochlaena rigidifolia
Mesosetum loliiforme
Mesosetum cayennense
Arthropogon filifolius
Arthropogon villosus
Altoparadisium chapadense
Achlaena piptostachya
Oplismenopsis najada
Phanopyrum gymnocarpon
Coleataenia petersonii
Coleataenia prionitis
Triscenia ovina
Coleataenia stenodes
Coleataenia caricoides
Coleataenia anceps
Coleataenia tenera
Coleataenia longifolia
Canastra lanceolata
Canastra aristella
Apochloa animarum
Apochloa euprepes
Apochloa cipoense
Apochloa steyermarkii
Apochloa subtiramulosa
Apochloa poliophylla
Apochloa molinioides
Apochloa lorea
Apochloa chnoodes
Arthropogoninae
Gerritea pseudopetiolata
Ichnanthus vicinus
Ichnanthus pallens
Ichnanthus nemorosus •
Ichnanthus panicoides
Echinolaena inflexa
Ocellochloa latissima
Ocellochloa rudis
Ocellochloa piauiensis
Ocellochloa chapadensis
Ocellochloa pulchella
Ocellochloa stolonifera
Ocellochloa irregularis
Ocellochloa craterifera
Ocellochloa biglandularis
Streptostachys lanciflora
Streptostachys asperifolia
Axonopus hydrolithica
Axonopus anceps
Axonopus singularis •
Axonopus fissifolius
Panicum venezuelae
Echinolaena standleyi
Echinolaena minarum
Renvoizea vaginiviscosa
Renvoizea trinii
Renvoizea sacciolepoides
Renvoizea restingae
Renvoizea acicularifolia
Renvoizea marauense
Osvaldoa valida
Aakia tuerckheimii
Anthaenantiopsis rojasiana
Hopia obtusa
Paspalum dasypleurum
Paspalum vaginatum
Paspalum distichum
Paspalum pygmaeum •
Paspalum conjugatum
Paspalum nutans
Paspalum dilatatum
Paspalum quadrifarium
Spheneria kegelii
Reimarochloa acuta
Paspalum malacophyllum
Paspalum erianthum
Paspalum virgatum
Thrasyopsis juergensii
Paspalum bertonii
Paspalum remotum
Paspalum conspersum
Paspalum glaziovii
Paspalum foliiforme
Paspalum haumanii
Paspalum wettsteinii
Paspalum arundinellum
Paspalinae
~100
~320
Outgroups
Thysanolaeneae
Cyperochloeae
Centotheceae
Tristachyideae
Chasmanthieae
Zeugiteae
Gynerieae
Arundinelleae
Andropogoneae
Paspaleae

Fig. S2b

Fig. S2a

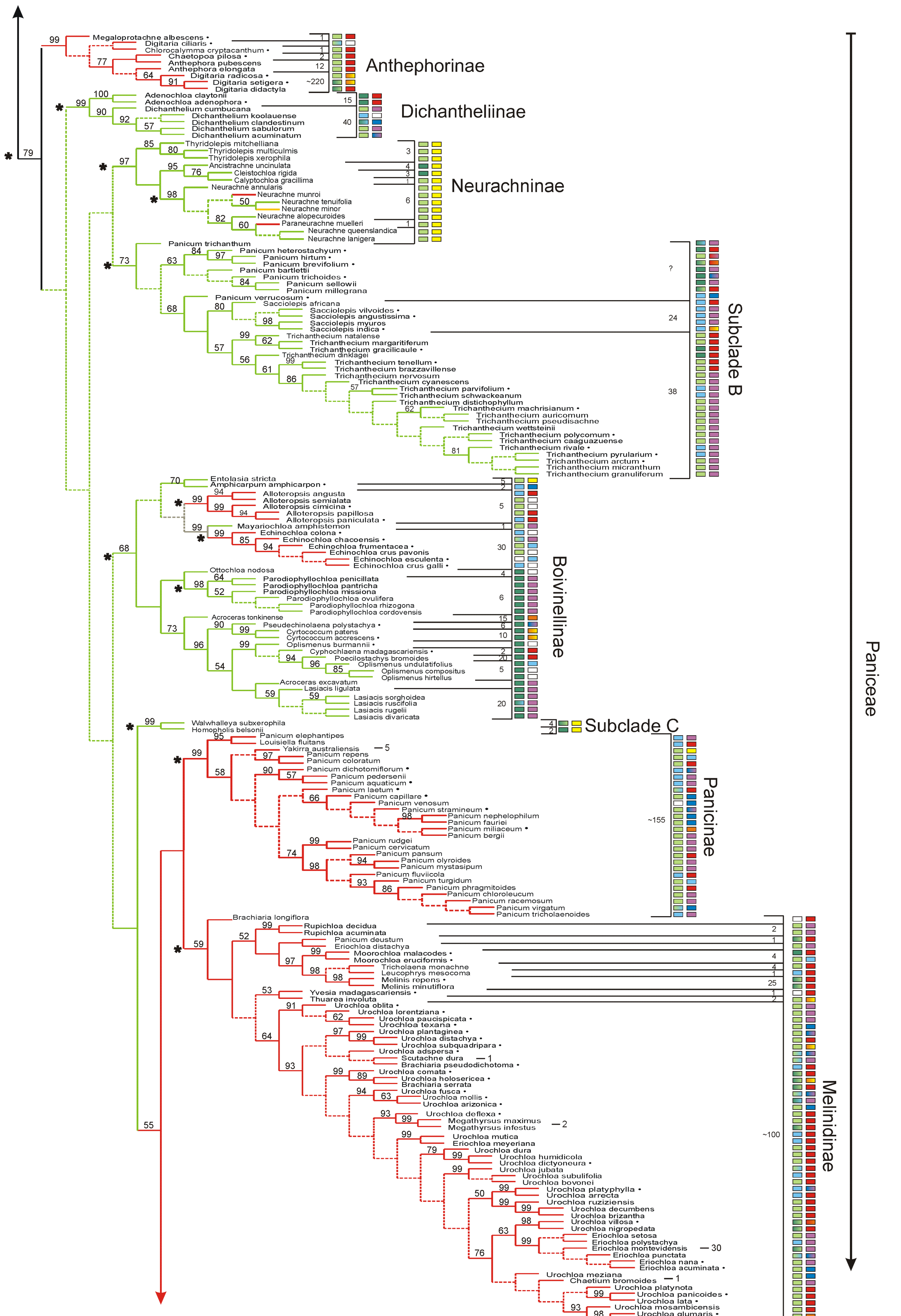

Fig. S2c

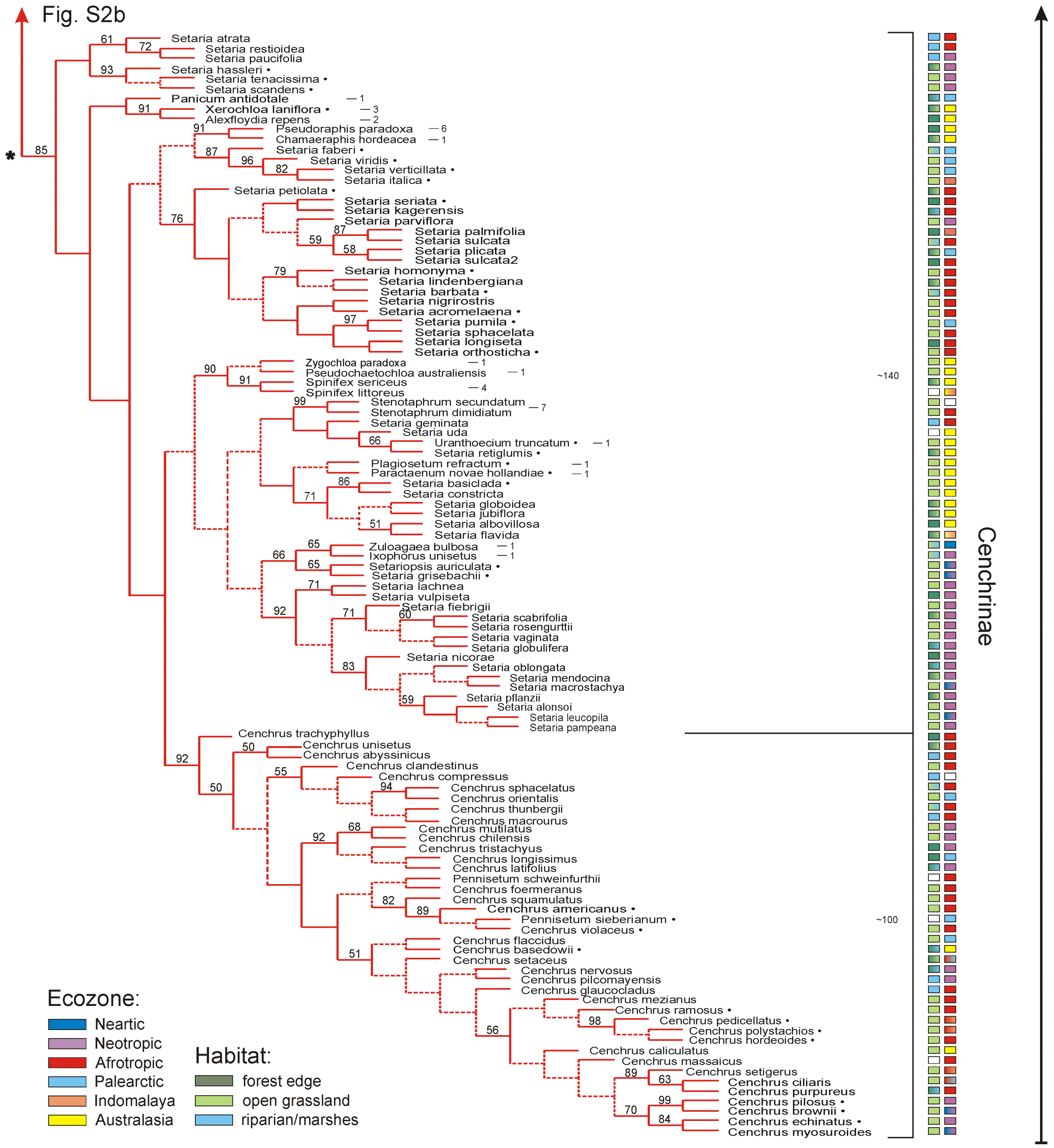

Figure S2. A selected optimal tree from the phylogenetic analysis showing jackknife values (10.000 replicates) and branches that are collapsed in the strict consensus tree (broken lines). Annual species are marked with a dot. The photosynthetic pathway is optimized on the tree (C_3 green, C_4 red, C_3-C_4 intermediate yellow). The optimization is only suggestive on numbers of C_4 origins as variations in the details of the C_4 photosynthesis within and among clades suggest a more complicated evolution of C_4 photosynthesis (Christin et al. 2012a,b). The estimated numbers of species in each genus are shown to illustrate undersampling of several clades. Clades marked with asterisks denote groups that were retained in the working phylogeny used in the PGLS regression (Table 1-2).
